# Crossed Cerebellar Atrophy of the Lateral Cerebellar Nucleus in an Endothelin-1-Induced, Rodent Model of Ischemic Stroke

**DOI:** 10.3389/fnagi.2017.00010

**Published:** 2017-02-17

**Authors:** Hugh H. Chan, Jessica L. Cooperrider, Hyun-Joo Park, Connor A. Wathen, John T. Gale, Kenneth B. Baker, Andre G. Machado

**Affiliations:** ^1^Department of Neurosciences, Lerner Research Institute, Cleveland ClinicCleveland, OH, USA; ^2^Center for Neurological Restoration, Cleveland ClinicCleveland, OH, USA

**Keywords:** crossed cerebellar diaschisis, crossed cerebellar atrophy, stroke, lateral cerebellar nucleus, corticopontocerebellar projections

## Abstract

Crossed cerebellar diaschisis (CCD) is a functional deficit of the cerebellar hemisphere resulting from loss of afferent input consequent to a lesion of the contralateral cerebral hemisphere. It is manifested as a reduction of metabolism and blood flow and, depending on severity and duration, it can result in atrophy, a phenomenon known as crossed cerebellar atrophy (CCA). While CCA has been well-demonstrated in humans, it remains poorly characterized in animal models of stroke. In this study we evaluated the effects of cerebral cortical ischemia on contralateral cerebellar anatomy using an established rodent model of chronic stroke. The effects of cortical ischemia on the cerebellar hemispheres, vermis and deep nuclei were characterized. Intracortical microinjections of endothelin-1 (ET-1) were delivered to the motor cortex of Long Evans rats to induce ischemic stroke, with animals sacrificed 6 weeks later. Naive animals served as controls. Cerebral sections and cerebellar sections including the deep nuclei were prepared for analysis with Nissl staining. Cortical ischemia was associated with significant thickness reduction of the molecular layer at the Crus 1 and parafloccular lobule (PFL), but not in fourth cerebellar lobule (4Cb), as compared to the ipsilesional cerebellar hemisphere. A significant reduction in volume and cell density of the lateral cerebellar nucleus (LCN), the rodent correlate of the dentate nucleus, was also noted. The results highlight the relevance of corticopontocerebellar (CPC) projections for cerebellar metabolism and function, including its direct projections to the LCN.

## Introduction

Diaschisis is defined as a loss of neural function due to reduced afferent input from a distant, but functionally connected, brain region that has been damaged by pathology (Finger et al., 2004; Machado and Baker, 2012). Crossed cerebellar diaschisis (CCD), which refers specifically to a loss of function affecting the hemicerebellum contralateral (i.e., contralesional) to a cerebral lesion, has been reported in Alzheimer’s disease (Akiyama et al., 1989), Lewy body dementia (Tatsch et al., 2003) and stroke (Tien and Ashdown, 1992; De Reuck et al., 1997; Takasawa et al., 2002; Komaba et al., 2004). CCD was also detected in a pediatric case in which the cerebellar atrophy was induced by abnormal cerebral development (Gallini et al., 2006). It is hypothesized that lesions affecting the cerebral cortex reduce the excitatory input to the cerebellum via the cortico-ponto-cerebellar (CPC) pathway (Carrera and Tononi, 2014), resulting in reduced metabolism and blood flow (Gold and Lauritzen, 2002). When deafferentation is chronic, it contributes to permanent degeneration of the cerebellum, termed crossed cerebellar atrophy (CCA; Gold and Lauritzen, 2002; Jiménez-Caballero, 2012). Further, although the cellular details of CCA remains unclear, recent studies showed that CCA associates with Bax-dependent apoptotic like cell death mechanism at the cerebellar cortex in both mouse and rat models of ischemia (Chiesa et al., 2005).

Several clinical studies have investigated CCA following ischemic stroke of the cerebral cortex in humans, with the volume of the contralesional cerebellum typically reported to be reduced relative to the unaffected, ipsilesional hemicerebellum (Tien and Ashdown, 1992; De Reuck et al., 1997; Takasawa et al., 2002; Komaba et al., 2004). Very few studies, however, have examined how these atrophic changes are distributed across the cerebellar cortex or deep cerebellar nuclei (Chung, 1985). Moreover, although CCD has been well-studied in rodent stroke models (Serteser et al., 2001; Gold and Lauritzen, 2002; Taylor et al., 2006), atrophic changes have largely been overlooked. Given the widespread use of rodent models of stroke in the development of clinical therapies and the association between CCD and functional outcomes, we characterized anatomical changes across the cerebellar cortex and deep nuclei induced by stereotactic, cerebral cortical endothelin-1 (ET-1) injections in a commonly-used rodent model of cerebral ischemia. ET-1 is a potent, long-acting peptide that induces dose-dependent localized vasoconstriction, with reperfusion occurring gradually over several hours post-injection. We examined the cerebellar cortex, including Crus 1 of the ansiform, the parafloccular lobule (PFL), the fourth cerebellar lobule (4Cb) in the vermis, as well as in the lateral cerebellar nucleus (LCN; the rodent homolog of the human dentate nucleus). As the LCN is a major nodal point within the CPC pathway, its pathological changes in the condition of CCA can confirm the current hypothesis of CCD/CCA formation. As the origin of ascending cerebellothalamocortical pathway, termed the dentatothalamocortical (DTC) pathway in humans, we have hypothesized previously that the LCN may be a potential target to reverse cortical dysfunction following cerebral cortical insults (Machado and Baker, 2012). In the present work, we hypothesized that cortical ischemia induced in the primary motor cortex would result in volume loss not only of the lateral cerebellar cortical layers, including the molecular and internal granule layers, but also within the LCN, resulting in subsequent CCA.

## Materials and Methods

### Animals

Ten male Long Evans rats, weighing 200–224 g at study onset, were housed on a 12:12 light/dark cycle. Food and water were available *ad libitum*. All experiments were conducted under a protocol approved by the Institutional Animal Care and Use Committee of the Cleveland Clinic.

### Surgery

Focal ischemia was induced as detailed previously (Cooperrider et al., 2014). Briefly, five rats were anesthetized with ketamine (50 mg/kg) and dexmedetomidine (0.5 mg/kg), fixed in a stereotaxic frame (David Kopf Instruments, Tujunga, CA, USA), and a craniotomy was performed over the sensorimotor cortex of the left cerebral hemisphere. Ischemia was induced by six intracortical injections of 800 pmol/2μl ET-1 (Millipore, MA, USA) at each of six stereotaxic coordinates in relation to bregma: (1) AP: −1.0, ML: +2.5, DV: −2.3; (2) AP: +1.0, ML: +2.5, DV: −2.3; (3) AP: +3.0, ML: +2.5, DV: −2.3; (4) AP: −1.0, ML: +3.5, DV: −2.3; (5) AP: +1.0, ML: +3.5, DV: −2.3; and (6) AP: +3.0, ML: +3.5, DV: −2.3 (Paxinos and Watson, 1998). After the injections were complete, the craniotomy was covered with cellulose paper (Data Sciences International) and tissue adhesive (Vetbond, 3M) to form a protective seal. Dexmedetomidine anesthesia was reversed with atipamezole (1 mg/kg), followed by buprenorphine (0.05 mg/kg, sc) administration for prophylactic pain management. The animals were monitored until fully recovered and returned, thereafter, to their home cages where food and water was provided *ad libitum*. Naïve control animals (*n* = 5) were used, as it is our experience that vehicle (i.e., saline injected) controls do no exhibit behavioral changes or cortical damage (Data not shown).

### Tissue Preparation

Six weeks after stroke induction, rats were anesthetized with 50 mg/kg sodium pentobarbital followed by decapitation. Brains were removed and fixed in 4% phosphate buffered paraformaldehyde (EMS, Hatfield, PA, USA) for 3 days. After cryoprotection in 30% sucrose/PBS, brain blocks were snap-frozen and preserved at −80°C until slicing by cryostat. Thirty micrometer sections of cerebral cortex and cerebellum were mounted on polysine-coated slides.

### Nissl Staining for Stroke Lesion Volume at the Motor Cortex and Size of the LCN

Cerebral and cerebellar sections were first stained with 0.4% cresyl violet in acidic PBS for 20 min after rehydrating with distilled water for 2 min. Cresyl violet staining was then destained by 70% ethanol in 1% acetic acid for 1 min. After dehydration with a series of ascending concentrations of ethanol and defatting with HistoClear, stained sections were mounted and coverslipped. Slices spanning the sensorimotor cortex as well as the cerebellum were mounted for analysis. Specifically, we mounted cortical sections corresponding to coordinates 4.2 mm anterior to bregma through 1.8 mm posterior to bregma, while coordinates for cerebellar sections were from 10.92 to 11.52 mm posterior to bregma (Paxinos and Watson, 1998). Stroke volumes were calculated as previously described by our group, utilizing a semi-automated method, SLICE (Park et al., 2013). Briefly, the lesion area of each Nissl-stained section was measured and the volume estimated by interpolation between the sections. The stroke lesions were then three-dimensionally projected onto the stereotactic atlas of the rat brain (Paxinos and Watson, 1998). Measurements were taken from both the stroke and naïve groups, despite the lack of significant change anticipated in the latter group.

The volume of the LCN (−10.92 mm to −11.52 mm to bregma), both ipsilesional and contralesional, was calculated using similar techniques. In order to be consistent with the stroke animals, the right LCN of the naïve cohort was defined as the index (pseudo-contralesional) location for the purpose of percent change calculations. Differences in LCN volume between the two hemispheres were calculated with the following equation:

$$\text{ABS}\left[\frac{\left(\text{Contralesional}\;\text{volume}\;-\;\text{Ipsilesional}\;\text{volume}\right)}{\text{Ipsilesional}\;\text{volume}}\times 100\%\right]$$

### Thickness of the Granular and Molecular Layers of Cerebellar Cortex

The highly-packed nature of the cerebellar granule neurons at the cerebellar cortex makes quantification of cell density impossible. Instead, we quantified the thickness of the internal granule and molecular layers of Crus 1 of the ansiform (Crus 1) and PFL, both of which are located in the lateral cerebellum and the 4Cb, located in the vermis, as an index of cerebral cortical ET-1 induced changes. Under 100× magnification, six measurements of thickness, each 20 μm apart, were taken using ImageJ (RRID: SCR_003070, NIH) for each lobule, both contralesional and ipsilesional. Areas selected for measurement are shown in Figure 2A. The thickness of the cerebellar lobules was examined from −10.92 mm to −11.52 mm in the anterior/posterior plane (in relation to bregma). Data were expressed as mean thickness (μm) ± SEM and the change (%) in thickness was calculated with the following equation:

$$\begin{array}{l} \text{ABS}\left[\frac{\left(\text{Contralesional}\;\text{thickness}\;-\text{Ipsilesional}\;\text{thickness}\right)}{\text{Ipsilesional}\;\text{thickness}}\;\times 100\%\right] \end{array}$$

As above, data from the right cerebellar hemisphere of naïve animals were operationally defined as the “contralesional” side for the purpose of this calculation.

### TUNEL Analysis

In order to evaluate apoptosis in the perilesional cerebral cortex, the cerebellar cortex and the LCN, cells were analyzed with a TUNEL assay kit (S7111, Millipore, MA, USA). Sections initially were incubated with TdT enzyme, which links digoxigenin-dNTP to apoptotic DNA fragments. Anti-digoxigenin antibody conjugated with fluorescein was applied to detect the digoxigenin-dNTP tails. After TUNEL assay, sections were counterstained with fluorescent Nissl dye (Life technologies, Carlsbad, CA, USA). TUNEL+/Nissl+ cells in the selected regions were counted by a blinded investigator at 200× magnification. The data were expressed as the density of TUNEL+/Nissl+ cells in a given area ± SEM.

### Quantification of Cell Numbers in the LCN

To characterize cell loss in the LCN, the contralesional and ipsilesional LCN of each Nissl-stained cerebellar section was highlighted at ×100 magnification and the area of the LCN was measured by ImageJ software (NIH). Thereafter, the number of Nissl+ cells in the LCN was quantified by the same program. Density of Nissl+ cells in the highlighted LCN was calculated with the following equation:

$$\text{Density}\;\text{of}\;\text{Nissl}\;\text{Cells}\;\text{=}\;\frac{\left(\text{Number}\;\text{of}\;\text{Nissl​+}\;\text{cells}\right)}{\left(\text{Area}\;\text{of}\;\text{the}\;\text{LCN}\;\text{in}\;{\text{μm}}^{2}\right)}$$

### Statistics

Comparisons of stroke volume and percentage reduction of internal granule and molecular layers were analyzed by Student’s *t*-test. Other comparisons were analyzed by one-way ANOVA with *post hoc* Tukey test using GraphPad Prism software (RRID: SCR_002798, GraphPad, La Jolla, CA, USA).

## Results

### Endothelin-1 Induced Stroke at the Motor Cortex

**Figure 1:**
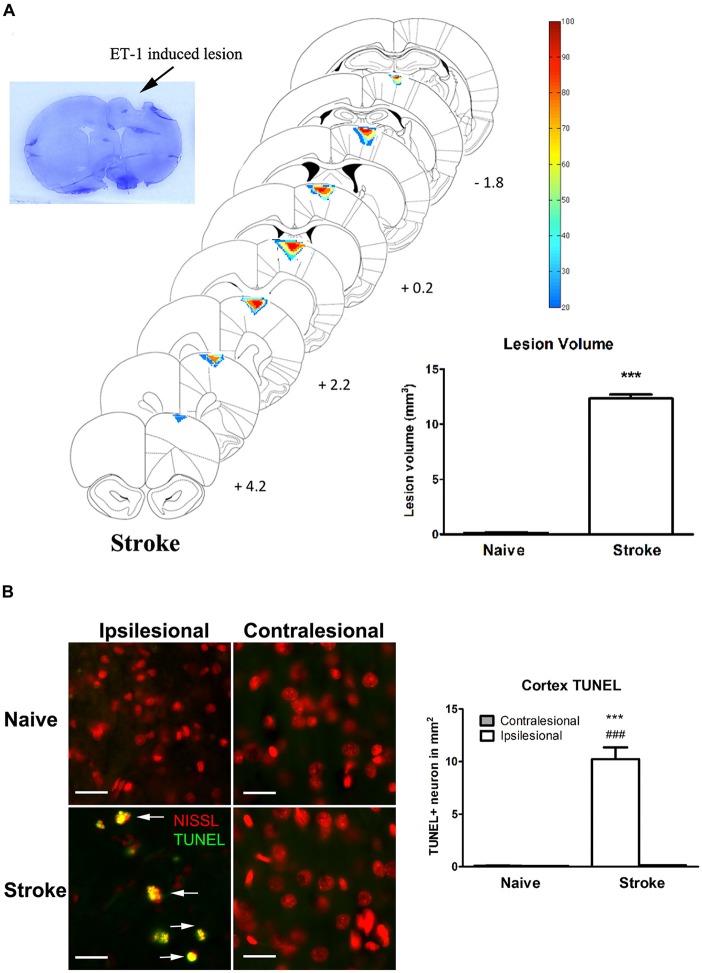
**Endothelin-1 (ET-1) induced stroke lesion at the motor cortex. (A)** Histological examination of stroke lesion location and volume. The stroke lesion was first visualized by Nissl staining. By using the SLICE program, stroke lesion location and volume are determined and overlaid on coronal sections from a rat atlas (Paxinos and Watson, 1998). The typical stroke spanned from 4.2 mm anterior to 1.8 mm posterior to bregma. Color coding represents the percentage of rats with lesioned tissue present at that pixel. The inset bar graph characterizes and compares the lesion volume between the naïve and stroke groups. Data are expressed as volume in mm^3^ ± SEM, analyzed by Student’s *t*-test. **(B)** ET-1 induced neuronal apoptosis at the ipsilesional motor cortex in stroke rats, which is not detectable at the contralesional side or in naïve rats (200x magnification). Neurons were stained with fluorescent Nissl in red. The density of TUNEL positive cells in the ipsilesional motor cortex of stroke rats was significantly higher than that in contralesional side and both contralesional and ipsilesional motor cortices of naïve rats (bar graph). Scale bar = 50 μm. Data are expressed as density of TUNEL positive cells in mm^2^ ± SEM, analyzed by one-way ANOVA with *post hoc* Tukey test. ***p < 0.0001 when compared to naïve group; ^###^*p* < 0.0001 when compared to the contralesional side of the same group.

ET-1 induced a significant lesion in the motor cortex of the stroke group (12.33 ± 0.18 mm^3^ vs. naïve: 0.194 ± 0.037 mm^3^, *p* < 0.0001, Figure 1A). Apoptotic cells (TUNEL+ cells) were observed in the perilesional area at a significantly higher rate than in the same topography on the contralesional motor cortex or in either cerebral hemisphere of the naïve group (Figure 1B). There was a significant increase of TUNEL+/Nissl+ cells in the ipsilesional motor cortex when compared to the contralesional motor cortex (One-way ANOVA: *F*_(3,82.78)_, *p* < 0.00001; Tukey’s test: ipsilesional: 10.23 ± 1.11 cells/mm^2^ vs. contralesional: 0.13 ± 0.03 cells/mm^2^; *p* < 0.0001, Figure 1B) and naïve group (naïve, left motor cortex: 0.098 ± 0.02 cells/mm^2^; *p* < 0.0001, Figure 1B).

### Endothelin-1 Induced Stroke Reduces the Thickness of Internal Granule and Molecular Layers at the Contralesional Cerebellar Cortex

**Figure 2:**
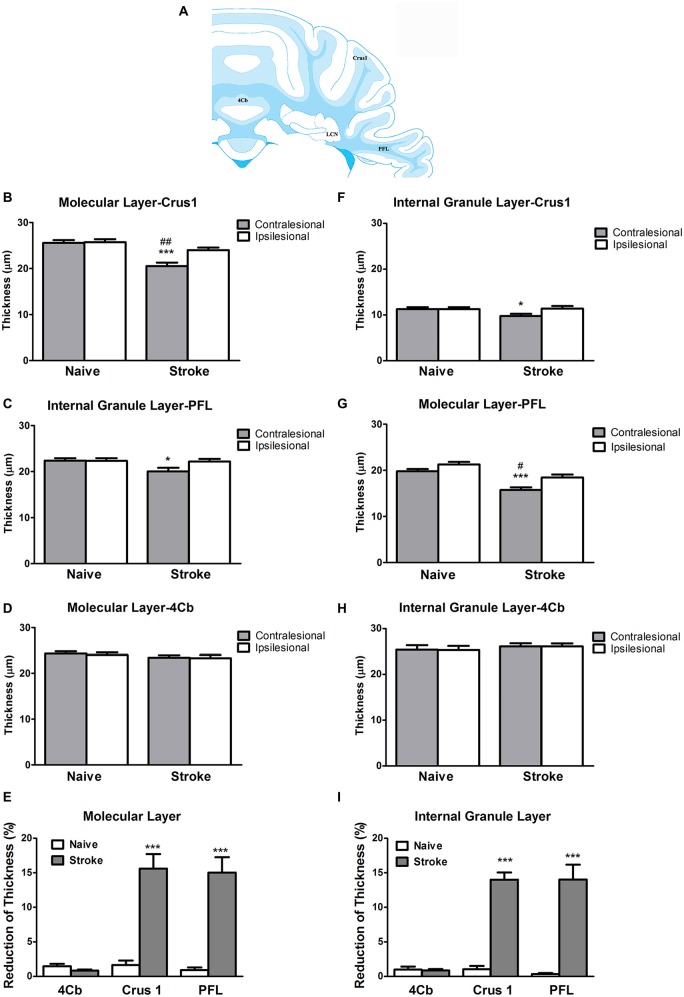
**ET-1 induced crossed cerebellar atrophy (CCA) at the cerebellar cortex. (A)** Schematic illustration of the area of quantification of thickness of molecular and internal granule layers of cerebellar cortex. ET-1 did not induce a reduction of thickness of the **(B)** internal granule and **(F)** molecular layers at the fourth cerebellar lobule (4Cb). However, there was a reduction of thickness of the **(C)** internal granule and **(G)** molecular layers of Crus1, also known as the ansiform lobule (**D**,**H** for paraflocculi, PFL), at the contralesional cerebella. Data are expressed as thickness (μm) ± SEM molecular or internal granule layers, analyzed by one-way ANOVA with *post hoc* Tukey test. The reduction of thickness of internal granule and molecular layers of Crus1, PFL and 4Cb were demonstrated as % reduction comparing to the ipsilesional side in (**E,I**), respectively, analyzed by Student’s *t*-test for each cerebellar cortical area. *p < 0.05 and ***p < 0.0001 when compared to naïve group; ^#^*p* < 0.05 and ^##^*p* < 0.01 when compared to ipsilesional side of the same group.

ET-1 administered at the cerebral cortex was associated with a significant reduction in the thickness of the contralesional cerebellar cortex. Specifically, there was a significant reduction in the thickness of the molecular layer at the Crus 1 (One-way ANOVA: *F*_(3,14.06)_, *p* < 0.0001; Tukey’s test, *p* < 0.0001; Figure 2B) and PFL (One-way ANOVA: *F*_(3,17.20)_, *p* < 0.0001, Tukey’s test, *p* < 0.0001; Figure 2C), but not 4Cb (One-way ANOVA: *F*_(3,0.70)_, *p* = 0.57, Tukey’s test, *p* = 0.24; Figure 2D) in the contralesional cerebellar cortex of stroke rats when compared to naïve rats. When compared to the ipsilesional sides of the same group, there were significant reductions in the thickness of the molecular at the Crus 1 (*p* < 0.01, Figure 2B) and PFL (*p* < 0.01, Figure 2C) in the stroke rats. However, there was no significant difference in 4Cb thickness (*p* = 0.88, Figure 2D). When comparing the percent reduction of thickness between the naïve and stroke groups the results were similar, with significant reductions of thicknesses of the molecular layer in Crus 1 (*p* < 0.0001, Figure 2E) and PFL (*p* < 0.0001, Figure 2E), but not in 4Cb (*p* = 0.33, Figure 2E).

At the level of the internal granule layer, comparing to the contralesional side of naïve rat, there was a significant reduction at the PFL (One-way ANOVA: *F*_(3,3.21)_, *p* < 0.05; Tukey’s test: *p* < 0.05; Figure 2G) but only a trend towards reduced thickness in Crus 1 that failed to reach significance (One-way ANOVA: *F*_(3,2.65)_, *p* = 0.08, Tukey’s test: *p* < 0.05; Figure 2F), but not 4Cb (One-way ANOVA: *F*_(3,0.27)_, *p* = 0.84, Tukey’s test, *p* = 0.57; Figure 2H). When comparing to the ipsilesional side of the same group, there were no significant reduction of thickness of internal granule layer in Crus 1 (*p* = 0.07, Figure 2F) and PFL (*p* = 0.06, Figure 2G). There was no difference detected in 4Cb (*p* = 0.97, Figure 2H). When comparing the percent reduction of internal granule layer thickness between stroke and naïve animals, there were significant reductions found in the Crus 1 (*p* < 0.0001; Figure 2I) and PFL (*p* < 0.0001; Figure 2I) but not 4Cb (*p* = 0.26, Figure 2I). No TUNEL+ cells were detected in either contralesional or ipsilesional cerebellar cortex of stroke animals.

### Endothelin 1-Induced Stroke Induces Reduction of Size of the LCN and Cell Density in the LCN

**Figure 3:**
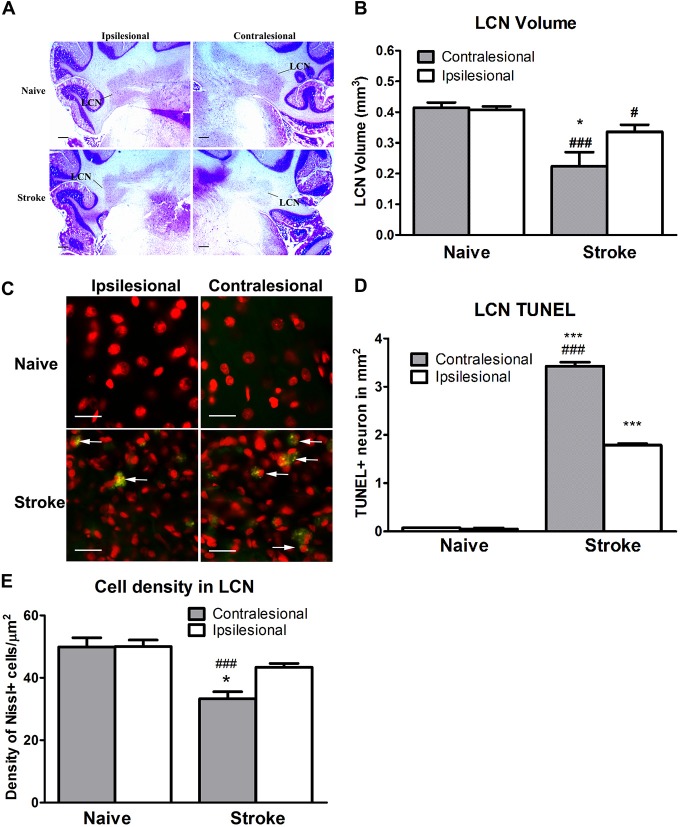
**ET-1 induced CCA at the lateral cerebellar nucleus (LCN). (A)** Representative images illustrating the LCN (annotated) at −11.0 mm in relation to bregma stained with cresyl violet. **(B)** ET-1 was associated with a reduction in LCN size in both contralesional and ipsilesional cerebellar hemispheres relative to naïve controls, with the reduction in the contralesional LCN being the larger of the two. Data are expressed as volume (μm^3^) ± SEM. **(C)** Representative images illustrating TUNEL positive neurons in the LCN. Neurons were stained with fluorescent Nissl in red (200x). **(D)** ET-1 induced apoptosis at both contralesional and ipsilesional LCN. Data expressed as density of TUNEL+ cells (in mm^2^) ± SEM. *p < 0.05 and ***p < 0.0001 when compared to the naïve group while ^#^*p* < 0.05 and ^###^*p* < 0.0001 when compared to the ipsilesional side of the same group. **(E)** ET-1 induced a reduction of both contralesional and ipsilesional LCN when compared to the naïve group, with the density decrease greater in the contralesional LCN. Data expressed as number of Nissl+ cells/mm^2^ ± SEM. Results in **(B,D,E)** are analyzed by one-way ANOVA with *post hoc* Tukey test. *p < 0.05 when compared to the ipsilesional LCN in the stroke group; ^###^*p* < 0.0001 when compared to the same side of LCN of the naïve group.

ET-1-induced cerebral ischemia was associated further with significant reductions in the size of both the contralesional and ipsilesional LCN (Figure 3A). The contralesional LCN was reduced by 46% in stroke relative to naïve animals (One-way ANOVA: *F*_(3,30.79)_, *p* < 0.0001; Tukey’s test: stroke: 0.22 ± 0.05 mm^3^ vs. naïve: 0.41 ± 0.02 mm^3^; *p* < 0.0001, Figure 3B), while the ipsilesional LCN volume within the stroke group was reduced by 17% (stroke: 0.34 ± 0.02 mm^3^ vs. naïve: 0.41 ± 0.01 mm^3^; *p* < 0.05, Figure 3B).

The reduction of the LCN volume was accompanied by apoptosis in stroke animals (Figure 3C). The density of TUNEL+/Nissl+ cells in both contralesional (One-way ANOVA: *F*_(3,11.53)_, *p* < 0.0001; Tukey’s test: *p* < 0.0001, Figure 3D) and ipsilesional (*p* < 0.0001, Figure 3D) LCN in the stroke group was significantly higher than in naïve animals. Likewise, a within-subjects comparison of TUNEL+/Nissl+ cell density in the stroke group revealed significantly higher than in the ipsilesional LCN (*p* < 0.0001, Figure 3D).

There was a significant difference in cell density between the contralesional and ipsilesional LCN of animals in the stroke group (One-way ANOVA: *F*_(3,12.45)_, *p* < 0.0001, Tukey’s test: *p* < 0.05, Figure 3E) that was not present in the naïve group (*p* = 0.97, Figure 3E). Furthermore, there was a significant reduction in Nissl+ cell density at the contralesional LCN of the stroke group when compared to that of naïve (*p* < 0.0001, Figure 3E). This finding was consistent with the reduction of LCN volume.

## Discussion

The anatomical and functional impact of disease or injury within the central nervous system is seldom confined to the region of primary insult, with interconnected areas throughout the brain and spinal cord displaying secondary functional, metabolic or structural anomalies. CCA as a result of chronic injury or degeneration of the cerebral hemispheres has been observed in humans (Tien and Ashdown, 1992; De Reuck et al., 1997; Komaba et al., 2004); however only a single case study has described subcortical changes involving the cerebellar dentate as the result of an arteriovenous vascular malformation in the cerebral cortex (Chung, 1985). Similarly, although several studies have characterized CCD in the rodent cerebellar cortex (Serteser et al., 2001; Takuwa et al., 2013), little is known about the impact of cerebral damage on subcortical cerebellar structures and underlying mechanisms. To our knowledge, this is the first report characterizing CCA as a function of specific cortical and subcortical cerebellar sub-regions in response to cerebral cortical ischemic injury in the rodent.

In the present study, atrophic changes were not uniform across the cerebellum, as differential effects were observed across the cerebellar cortex. Specifically, we observed a reduction in cortical thickness predominantly on the internal granule and molecular layers of the lateral, but not medial, cerebellar cortex. Interestingly, these changes were not restricted to the contralateral cerebellar hemisphere, however the magnitude of degeneration in both cortical and subcortical structures was markedly greater in the contralesional hemisphere. In addition, we showed that after cortical ischemia there is a significant loss of LCN volume accompanied by markers of neuronal apoptosis (Taylor et al., 2006). However, we did not detect apoptotic cells in the cerebellar cortex. Elevations of nitric oxide level and excitotoxicity in the contralesional cerebellar cortex following MCAO ischemic lesions have been reported (Balkan E. et al., 1997; Balkan S. et al., 1997; Serteser et al., 2001), however no direct evidence of apoptosis as the underlying degenerating mechanism was illustrated. The absence of apoptosis in the cerebellar cortex under the condition of CCA may indicate that the excitotoxic cell loss in both granule and molecular layers are not via programmed cell death mechanisms (Üçal et al., 2017). Alternatively, it is possible that there is CCA-associated apoptosis at the cerebellar cortex but that apoptotic markers cannot be detected 6 weeks after ischemic induction. Further apoptotic assays and investigation at different time points should be adopted in future studies. Moreover, studying the cell death mechanism of the LCN neurons, which comprise a mixture of glutamatergic and GABAergic neurons with different morphological characteristics, could elucidate the role of excitotoxicity induced by different pathways, including the CPC, innervating the LCN. This might include examining the neuronal degeneration in the LCN using dual immunohistochemistry of apoptotic (or non-apoptotic) and glutamatergic (or GABAergic) markers.

### Cortico-Ponto-Cerebellar Pathway

Denervation of the CPC pathway leads to a reduction of excitatory input to the cerebellum and is the etiological factor thought to contribute to reduced neural activity along with diminished blood flow and, ultimately, neuronal death in the contralesional cerebellar cortex (Taylor et al., 2006; Gold and Lauritzen, 2002 #33; Jiménez-Caballero, 2012; Machado and Baker, 2012). The pontine nucleus primarily targets contralateral cerebellar structures; with projections diverging towards both the cerebellar cortex (Brodal and Steen, 1983; Brodal and Bjaalie, 1997; Xiong et al., 2002) and the deep cerebellar nuclei, including the LCN (Shinoda et al., 1992, 1993; Mihailoff, 1994). This distribution leaves both cortical and subcortical regions subject to deafferentation and degeneration in response to reduced cerebral input. At the level of the cerebellar cortex, we observed changes predominantly in more lateral cortical regions, with no significant reduction in the thickness across either the molecular or internal granule layers of 4Cb. This differential impact may simply reflect that, although the CPC pathway does project to the vermis (Xiong et al., 2002), the vast majority of its projections target the lateral cerebellum (Serapide et al., 2002). Volume changes in the LCN are similarly attributable to a loss of cortical input to the pontine nuclei. However given that the LCN also receives GABAergic Purkinje cell input (Tsubota et al., 2013) from the degenerating cerebellar cortex, the precise role of loss of excitatory (from the pontine nuclei) vs. inhibitory (from the cerebellar cortex) inputs on LCN degeneration is unclear. Further investigation of the afferent axons and synapses to the LCN is necessary to clarify the extent of contribution from each mechanism. Finally, while the atrophic changes were found to be more profound in the cerebellar hemisphere opposite the unilateral cerebral infarct, similar, albeit more modest, changes in the ipsilateral cerebellum likely underscore the bilateral nature of pontine projections (Cicirata et al., 2005). Notably, a limitation of the current study is that cerebral infarcts were generated only in the left hemisphere of animals and future studies should consider targeting each cerebral hemisphere in order to confirm that pattern of CCA reverses.

A more complete characterization of CCD/CCA pathology will help better understand long-term clinical findings after cortical ischemia and will be relevant to the development and interpretation of future preclinical experiments (Carrera and Tononi, 2014). Furthermore, the presence of CCD has been shown to limit the prognosis of post-stroke rehabilitation (Takasawa et al., 2002), suggesting that therapies should be aimed not only at facilitating changes in the perilesional cortex but also at reversing diaschisis (Machado and Baker, 2012). Following injury, the infarcted cerebral cortex may be re-organized under the influence of excitatory input from the DTC pathway. However, if the DTC is impaired at its origin (the LCN) as a result of CCA, there will be limited excitatory input to the cortex, potentially limiting plasticity and recovery. Novel treatment approaches, including the upregulation of neural activity across the DTC pathway, may facilitate reorganization and recovery (Machado et al., 2009, 2013; Cooperrider et al., 2014; Park et al., 2015). Overall, our current findings support the need to better characterize the nature and extent of cerebellar changes in the cortex and deep nuclei in response to cerebral infarct, both with regards to the mechanistic underpinnings of CCD and CCA as well as the potential to explore these phenomena for the development of novel therapies.

## Author Contributions

HHC and JLC are responsible for experimental design, animal experiments, data collection and manuscript preparation. H-JP and CAW are responsible for the data acquisition, interpretation of data and manuscript preparation. JTG, KBB and AGM are responsible for the experimental design, interpretation of data and manuscript preparation. All authors approved the final draft of manuscript and endorsed the agreement statement.

## Funding

This research was funded by: The Charles and Christine Carroll Family Endowed Chair in Functional Neurosurgery, and National Institutes of Health (NIH; R01 HD061363).

## Conflict of Interest Statement

AGM is a consultant with Functional Neuromodulation, Spinal Modulation, Saint Jude. Distribution rights with Enspire, ATI and Cardionomics. Fellowship support from Medtronic. The other authors declare that the research was conducted in the absence of any commercial or financial relationships that could be construed as a potential conflict of interest.
